# Characterization of a Novel SARS-CoV-2 Genetic Variant with Distinct Spike Protein Mutations

**DOI:** 10.3390/v13061029

**Published:** 2021-05-29

**Authors:** Anna Gladkikh, Anna Dolgova, Vladimir Dedkov, Valeriya Sbarzaglia, Olga Kanaeva, Anna Popova, Areg Totolian

**Affiliations:** 1 Saint Petersburg Pasteur Institute, 197101 Saint Petersburg, Russia; angladkikh@gmail.com (A.G.); annadolgova@inbox.ru (A.D.); sbarzaglia.valeriya@gmail.com (V.S.); ol.kanaeva@gmail.com (O.K.); totolian@spbraaci.ru (A.T.); 2 Martsinovsky Institute of Medical Parasitology, Tropical and Vector Borne Diseases, Sechenov First Moscow State Medical University, 119991 Moscow, Russia; 3 Federal Service for Surveillance on Consumer Rights Protection and Human Well-Being, 127994 Moscow, Russia; anna.popova.00.00@mail.ru

**Keywords:** SARS-CoV-2, COVID-19, variants of concern, northwest (NW) variant, Russia

## Abstract

The COVID-19 pandemic, which began in Wuhan (Hubei, China), has been ongoing for about a year and a half. An unprecedented number of people around the world have been infected with SARS-CoV-2, the etiological agent of COVID-19. Despite the fact that the mortality rate for COVID-19 is relatively low, the total number of deaths has currently already reached more than three million and continues to increase due to high incidence. Since the beginning of the pandemic, a large number of sequences have been obtained and many genetic variants have been identified. Some of them bear significant mutations that affect biological properties of the virus. These genetic variants, currently Variants of Concern (VoC), include the so-called United Kingdom variant (20I/501Y), the Brazilian variant (20J/501Y.V3), and the South African variant (20H/501Y.V2). We describe here a novel SARS-CoV-2 variant with distinct spike protein mutations, first obtained at the end of January 2021 in northwest Russia. Therefore, it is necessary to pay attention to the dynamics of its spread among patients with COVID-19, as well as to study in detail its biological properties.

## 1. Introduction

More than a year has passed since the beginning of the COVID-19 epidemic, which occurred in late December 2019 in Wuhan, Hubei Province (China). Since that time, the epidemic has become a pandemic covering all continents, with the exception of Antarctica. As of the end of April 2021, 140,332,386 cases of COVID-19 have been identified, 3,004,088 of which were fatal (https://www.who.int/publications/m/item/weekly-epidemiological-update-on-covid-19---20-april-2021, accessed on 28 May 2021). Spread of the virus was facilitated by several factors: the airborne transmission route of SARS-CoV-2, the etiological agent of COVID-19, active cross-border migration of the population, and delays in the introduction of restrictive measures by a number of countries due to epidemic situation complications. 

Factors complicating the detection of SARS-CoV-2 infection include the similarity of COVID-19 clinical symptoms with other acute respiratory diseases and the presence or mild or asymptomatic forms of the disease. Such heterogeneous clinical symptoms (ranging from asymptomatic to acute respiratory failure) combined with a lack of specific diagnostic tools in the pandemic’s initial stage also contributed to rapid, widespread infection.

Russia shares an expansive border with China. In addition, the cross-border flow of Chinese and Russian citizens, before the outbreak of the pandemic, was about 6 million per year. However, timely anti-epidemic measures made it possible to delay the spread of SARS-CoV-2 for three months. The first COVID-19 patient in Russia was registered on 2 March 2020 [1]. It is noteworthy that the introduction of the virus to Russia occurred not from China, but from Europe; this led to the formation of a specific genetic profile of variants circulating in the country [1].

Currently, information about the genetic diversity of SARS-CoV-2 in Russia is restricted due to the relatively small number of sequences uploaded to available databases, such as NCBI GenBank or GISAID. Nevertheless, based on the information available at the beginning of February, the most common genetic variants in Russia are those belonging to the 20B clade, according to the GISAID database. There are also a small number of sequences attributed to 20H/501Y.V2 and 20I/501Y.V1 (https://www.gisaid.org/phylodynamics/russia/, accessed on 28 May 2021).

It is well known that the S, E, M, and N genes of SARS-CoV-2 encode structural proteins, while non-structural proteins (such as 3-chymotrypsin-like protease, papain-like protease, and RNA-dependent RNA polymerase) are encoded by the ORF 1a and ORF 1b regions [2]. The S protein consists of an extracellular N-terminus, a transmembrane (TM) domain anchored in the viral membrane, and a short intracellular C-terminal segment [3]. The S protein usually exists in a metastable prefusion conformation. When the virus interacts with a host cell, structural rearrangement of the S protein occurs, allowing the virus to fuse with the host cell membrane.

In this report, we describe two genomes sequenced during routine studies of the genetic diversity of strains circulating in the Northwestern Federal District of Russia. The sequences have pronounced genetic differences in the gene encoding the SARS-CoV-2 S protein. 

## 2. Materials and Methods

### 2.1. Sample Collection

During routine study of SARS-CoV-2 genetic diversity in Russia up to January 2021, 834 nasopharyngeal swabs from patients with COVID-19, admitted to hospitals located in different regions of northwest Russia, were collected and delivered to the Saint Petersburg Pasteur Institute for sequencing and further phylogenetic study. Swabs were collected in 500 µL of special transport medium or phosphate-buffered saline (pH 7.0) and stored at −20 °C until analysis.

### 2.2. RNA Extraction and Reverse Transcription qPCR

Total nucleic acid samples were obtained by extraction and purification using the RIBO-prep DNA/RNA Extraction Kit (AmpliSens^®^, Russia) according to the manufacturer’s recommendations. DNA/RNA was eluted with 50 µL of the elution buffer (AmpliSens^®^, Russia) and stored at −70 °C until molecular analysis. For SARS-CoV-2 detection and to assess concentration, nucleic acids from swabs were thoroughly analyzed using the COVID-19 Amp RT-qPCR Kit (Saint Petersburg Pasteur Institute, Russia) according to the manufacturer’s recommendations [4]. SARS-CoV-2-positive samples, featuring Ct values of 20 or less, were selected and studied further.

### 2.3. Primer Design for Near-Complete Genome Sequencing

In order to obtain near-complete genome sequences of SARS-CoV-2 strains (excluding the 5’ and 3’ ends), a total of 64 primer pairs were designed (Supplementary Table S1) using the Primal Scheme (http://primal.zibraproject.org, accessed on 28 May 2021) web-based primer design tool [5]. For SARS-CoV-2, we used amplicon lengths of about 550–600 nts with 50 nt overlaps. Sequence of the Wuhan-Hu-1 SARS-CoV-2 isolate was used as the reference genome (NCBI GenBank NC_045512.2).

### 2.4. Library Preparation and Near-Complete Genome Sequencing

Reverse transcription was performed using random hexanucleotide primers and the Reverta-L Kit (AmpliSens^®^, Russia) according to the manufacturer’s instructions; cDNA samples were stored at –70 °C and subsequently used as amplification templates. The designed primers were sorted into eight groups, each containing eight primer pairs. In result, eight groups of 550–600 bp DNA fragments were amplified that were suitable for subsequent 600-cycle sequencing by the Illumina MiSeq System (Illumina Inc., USA) (Table 1).

Hot-start multiplex PCR amplification reactions were performed in a 25 µL total volume containing 2 µL of template cDNA, 0.1 µM of each sense primer, 0.1 µM of each antisense primer, and 12.5 µL of 2x BioMaster HS-Taq PCR mix (BiolabMix, Novosibirsk, Russia). The following thermal cycling parameters were employed: 95 °C for 3 min, 40 cycles (93 °C for 10 s, 57 °C for 30 s, 72 °C for 30 s), and a final extension at 72 °C for 5 min. Reactions were performed in a C1000 Touch thermocycler (Bio-Rad, USA). Products were analyzed by 2.0% agarose gel electrophoresis in the presence of ethidium bromide.

Concentrations of the fragments were measured with a Qubit 2.0 fluorometer (Invitrogen, USA) using the Qubit dsDNA HS Assay Kit (Invitrogen, USA). Fragments were mixed equimolarly, cleaned by means of the QIAquick PCR Purification Kit (Qiagen, Germany) according to the manufacturer’s instructions, and then used for library preparation.

Libraries were prepared using the TruSeq Nano DNA Kit (Illumina Inc., USA) and the TruSeq DNA CD Indexes Kit (Illumina Inc., USA). Quality assessment of final libraries was carried out on the QIAxcel Advanced capillary system (Qiagen, Germany). Sequencing was performed using the Illumina MiSeq System (Illumina Inc., USA) with the MiSeq Reagent Kit v3 (600-cycle) (Illumina Inc., USA).

### 2.5. In Silico Analysis

#### 2.5.1. Genome Assembly

The quality of Illumina reads was assessed using the FastQC program [6]. Raw reads were filtered with Trimmomatic [5] to remove adapters, low-quality nucleotides, and biased sequences at the ends of the reads (parameters ILLUMINACLIP: TruSeq3-PE. fa: 2:30:10:2 SLIDINGWINDOW: 4:20 LEADING:3 TRAILING:3 MINLEN:36). Genome assembly was carried out by mapping to the SARS-CoV-2 reference genome (strain Wuhan-Hu-1, NCBI accession number NC_045512.2) using the Geneious Prime program [7]. For the assembly, five independent iterations were launched with the minimum genome coverage parameter not less than five. Genome annotation was performed based on the reference genome.

#### 2.5.2. Phylogenetic Reconstructions

Alignment of nucleotide sequences was performed in mafft v. 7.475 [8]. SNV search and analysis was performed using MEGA X software [9]. A phylogenetic tree was constructed using the tools implemented in Nextstrain custom builds (https://github.com/nextstrain/ncov, accessed on 28 May 2021) [10]. A test for probable recombination was performed using the Recombination Detection Program (RDP) 4 beta 80 using eight methods provided by the software and default settings [11].

#### 2.5.3. Protein Analysis

Sequences were aligned and their consensus or identical aa residues were determined by Vector NTI Advance 11.0 (Invitrogen, USA) [12]. The 3D structure was predicted by SWISS-MODEL [13].

## 3. Results

### 3.1. Sequencing

Among the sequences obtained, two have distinct mutations in the spike glycoprotein gene, specifically: a 27-nucleotide deletion at positions 21,967-21,993 in the reference genome (Wuhan-Hu-1 strain, NCBI GenBank accession number NC_045512.2) and a 12-nucleotide insertion at positions 23,598-23,599 in the reference genome. Both sequences carried the deletion and the insertion. The first sequence (isolate SPb-117) was obtained from an unvaccinated patient in Saint Petersburg, a 20-year-old woman with symptoms such as fever (37.7 °C), weakness, and rhinitis. She had not traveled recently but did have contact with a COVID-19 patient. The swab was collected on 22 January 2021. The second sequence (isolate P-16) was obtained from an unvaccinated 32-year-old man with symptoms such as fever (38.5 °C), headache, shortness of breath, anosmia, and weakness. The swab was collected on 18 January 2021.

Sequencing produced 125,338 and 158,616 paired reads for SPb-117 and P-16 samples, respectively. After trimming, 94,817 and 120,390 paired reads were mapped to the Wuhan-Hu-1 reference genome. The mean coverages were 1,270 for isolate SPb-117 and 1,932 for isolate P-16. The sequences were designated hCoV-19/Russia/SPb-117/2021 and hCoV-19/Russia/Pskov-16/2021. Both sequences were annotated and submitted to NCBI GenBank (accession numbers MW750605, MW750606) as well as to GISAID (accession numbers EPI_ISL_1259282, EPI_ISL_1259283). Taking into account the uniqueness of the identified genetic features as well as the localization of the identified isolates in the northwest of Russia, we designated these sequences as the northwest variant of SARS-CoV-2 (NW variant).

### 3.2. Phylogenetic Analysis

Pairwise comparison of complete/near-complete nucleotide sequences showed that the NW variants share maximum nucleotide identity (99.71–99.82%) with the genome of SARS-CoV-2 hCoV-19/Qatar/QA-WCMQ_FD18163187/2020 (GISAID accession number EPI_ISL_1714455). The sequence was obtained in Qatar from a sample collected on August 10, 2020. In addition, pairwise comparison based on S-gene nucleotide sequences showed that the NW variants share maximum nucleotide identity (99.40–99.45%) with the genome of SARS-CoV-2 hCoV-19/USA/GA-CDC-LC0029877/2021 (GISAID accession number EPI_ISL_1462645). The sequence was obtained in the United States from a sample collected on 16 March 2021. 

**Figure 1:**
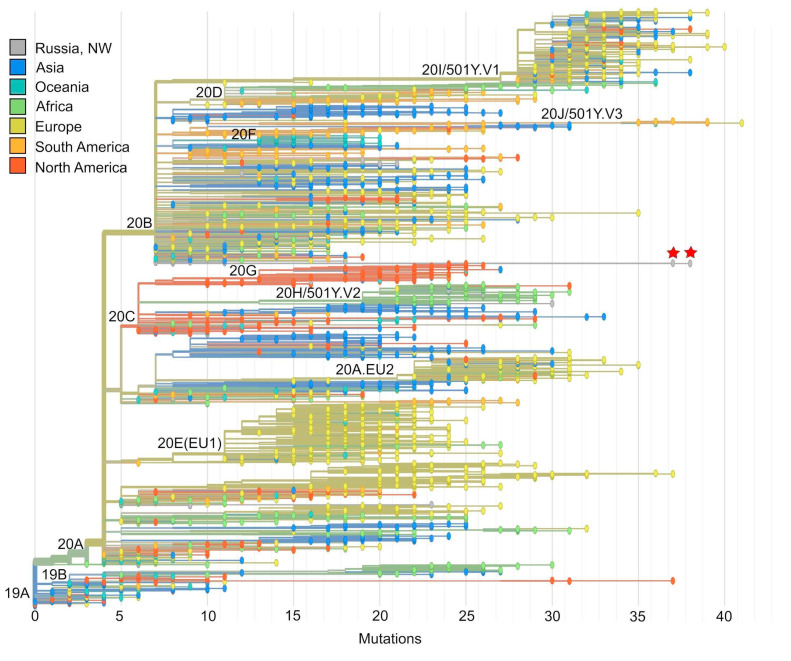
Phylogenetic tree reconstruction based on Nextstrain tools. Strains belonging to the northwest (NW) variant of SARS-CoV-2 (hCoV-19/Russia/SPb-117/2021, MW750605 and hCoV-19/Russia/Pskov-16/2021, MW750606) form a separate branch within the 20B clade, according to Nextstrain nomenclature (marked by red stars).

According to different classification nomenclatures, the NW sequences belong to clade 20B, according to Nextstrain [10]; clade GR, according GISAID; or lineage AT.1 (alias of B 1.1.370.1), according to PANGOLIN (Phylogenetic Assignment of Named Global Outbreak LINeages) [14]. On the Nextstrain-based tree, they form a separate, long branch within clade 20B (Figure 1). No recombination events were detected in isolates SPb-117 or P-16 using RDP 4 software.

**Figure 2:**
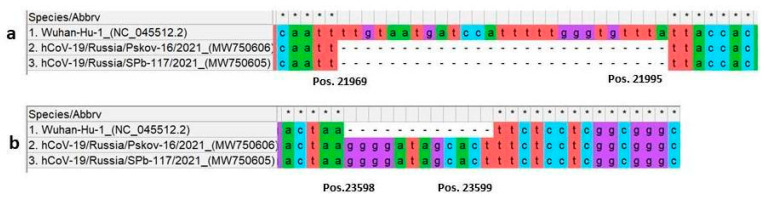
Northwest (NW) variant-specific mutations in the viral spike protein. Sequences were aligned using MEGA X software [9]. The sequence of SARS-CoV-2 Wuhan-Hu-1 (NCBI GenBank accession number NC_045512.2) was used as the reference. (**a**) Location of 27 nt deletion (in both NW variant sequences obtained); (**b**) location of 12 nt insertion (in both NW variant sequences obtained).

Pairwise comparison of the NW variant genomes with the Wuhan-Hu-1 reference genome (NCBI GenBank accession number NC_045512.2) enabled identification of a number of features. In addition to synonymous and nonsynonymous substitutions, these included a deletion (21969DEL21995, Figure 2a) and an insertion (23598IN23599, Figure 2b) in both NW isolates (SPb-117, P-16, Table 1). Some mutations observed, including indels, occurred in the viral spike-protein gene.

### 3.3. Protein Analysis

The distinctive features of the SARS-CoV-2 NW variant described in this article are the deletion of nine amino acids C136_Y144del (CNDPFLGVY) and the insertion of four amino acids N679delinsKGIAL in the spike-glycoprotein gene (relative to the Wuhan-Hu-1 reference genome). 

The total length of the NW variant’s spike glycoprotein was 1268 amino acid residues (1273 aa in Wuhan-Hu-1), with subunits as follows: a signal peptide (1–13 aa), S1 subunit (14–680 aa), and S2 subunit (681–1268 aa). The S1 subunit has an N-terminal domain (14–296 aa) and a receptor-binding domain (RBD, 310–532 aa). The S2 subunit is composed of a fusion peptide (FP, 783–801 aa), a heptapeptide repeat 1 sequence (HR1, 907–979 aa), HR2 (1158–1208 aa), a transmembrane domain (TM-domain, 1208–1232 aa), and a cytoplasmic domain (1233–12368 aa). Domain locations were determined in accordance with the reference aa sequence of SARS-CoV-2 Wuhan-Hu-1 (NCBI GenBank accession number NC_045512.2) [15]. 

## 4. Discussion

**Figure 3:**
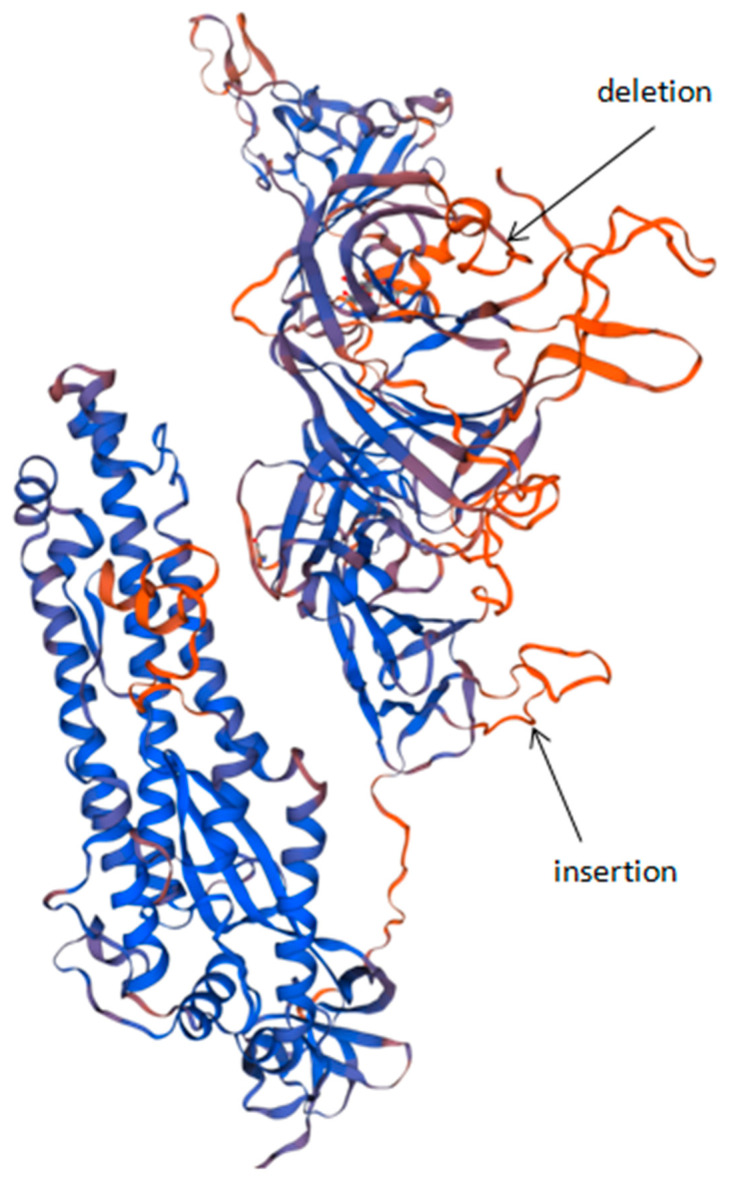
Structural model of variant SARS-CoV-2 S protein, SPb-117 strain (NW), based on PDB:7cwu.1 structure [24]. Black arrows indicate the positions of the main mutations of the described strain: the deletion of nine amino acids, C136_Y144del (Wuhan-Hu-1 strain numbered residues); and the insertion of four amino acids, N679delinsKGIAL. Both mutations lie in protruding regions of the amino acid chain.

A distinctive feature of the NW variant is a difference in the S protein’s amino acid composition. Changes in the described sequences do not critically affect the overall structure of the protein. The S protein’s three-dimensional structure was predicted using the Wuhan-Hu-1 strain protein model. In Figure 3, the location of the insertion and the deletion, in accordance with the three-dimensional structure, are visible. On the 3D model, the locations of the 4 aa insertion and 9 aa deletion are marked. 

Generally, the place wherein insertion occurred forms an exposed loop that harbors multiple arginine residues (multibasic) [16,17]. There, the S proteins of all SARS-CoV-2 strains contain a cleavage site, RXXR, recognized by the cellular protease furin to separate the S1 and S2 subunits. In a vesicular stomatitis virus model carrying S protein, it was shown that replacement of the S1/S2 site in the original SARS-CoV-2 protein by mutant ones (similar to SARS and RaTG13) leads to the impossibility of its cleavage. Arginine supplementation did not significantly affect protein activation by protease. 

**Figure 4:**
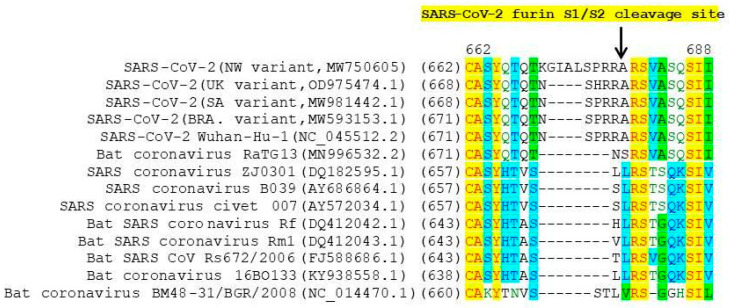
Amino acid alignment of betacoronaviruses in the region of furin S1/S2 cleavage site. Strictly conservative, identical, and similar residues are highlighted in yellow, blue, and green, respectively. SARS-CoV-2 furin cleavage site RXXR marked with an arrow. NW SARS-CoV-2 variant has a four-amino-acid insertion N679delinsKGIAL in comparison with Wuhan-Hu-1 strain.

This protease cleavage is necessary for promoting viral spread through cells of the human lung. In addition, using S proteins with altered cleavage sites, the researchers found that the S1/S2 site of SARS-CoV-2 is required for virus-induced fusion of infected cells with nearby cells and the formation of syncytium, and the additional arginine residue enhances fusion [18]. However, other betacoronaviruses do not have this cleavage site (Figure 4).

In the NW variant isolates obtained, an additional insertion of four amino acid residues (N679delinsKGIAL) is located directly before the cleavage site (Figure. 4) that is not present in other SARS-CoV-2 variants. It is possible that such a mutation may affect the efficiency of furin cleavage and, consequently, viral entry into the cell.

**Figure 5:**
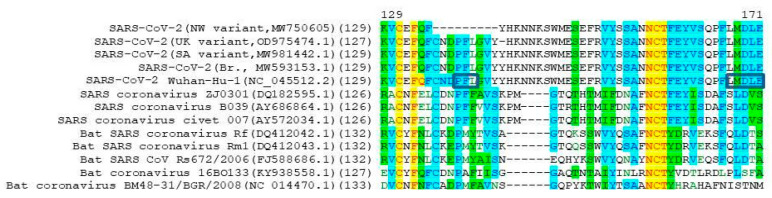
Amino acid alignment of betacoronaviruses in the region of deletion of NW SARS-CoV-2 variant. Strictly conservative, identical, and similar residues are highlighted in yellow, blue, and green, respectively. Declared nine amino acid deletions in NW variant are located in the position C136_Y144del of the Wuhan-Hu-1 strain. Position of a DP[FW] motive and clathrin box motif marked with frames on the sequence of Wuhan-Hu-1 strain.

Another distinctive and unique feature of the obtained NW variant isolates is a deletion of certain residues C136_Y144del. Inside this deletion, there is a DPF motif (138DPF140 in the Wuhan-Hu-1 reference strain) (Figure 5), which is defined by the ELM resource as a variation of a known motif, DP[FW] [19]. These motifs are responsible for the binding of accessory endocytic proteins to the alpha subunit of adaptor protein AP-2 and their recruitment to the site of clathrin-coated vesicle formation [20]. Clathrin-coated vesicles are responsible for a large fraction of the vesicular traffic that reaches the endosomal compartment, originating from the plasma membrane or from the TGN (trans-Golgi network). 

The assembly of the clathrin-coated vesicles is mediated by protein adaptors like AP (Adaptor Protein) complexes. The AP-2 complex is a heterotetramer consisting of two large adaptins (alpha and beta), a medium adaptin (mu), and a small adaptin (sigma). The beta subunit of the AP-2 complex binds to clathrin. The mu subunit interacts with the Y-based sorting signal present in the cytosolic tails of membrane receptors. Tyrosine-based signals fitting the YXXØ motif mediate sorting of transmembrane proteins to endosomes, lysosomes, and the basolateral plasma membrane of epithelial cells [21]. The alpha subunit of AP-2 binds regulatory/accessory proteins involved in the control of clathrin-coated vesicle formation [22,23]. 

For SARS-CoV, it was shown that, after its binding to ACE2, clathrin-coated pits are formed by interactions between the ACE2/virus complex and the AP2/clathrin complex via a possible coreceptor in a non-lipid-raft portion of the plasma membrane [24]. It was identified that the AP-2 mu subunit (AP2M1) is a crucial host factor for coronaviral entry and can be targeted by kinase inhibitors like sunitinib. AP2M1 interacts with the YASI sequence in the cytoplastic tail of ACE2 and mediates clathrin-dependent entry for SARS-CoV. Since SARS-CoV-2 also uses the ACE2 receptor, the function of AP2M1 in SARS-CoV-2 entry may be similar to that in SARS-CoV entry [25]. 

In 2021, a study appeared providing clear evidence that clathrin-mediated endocytosis is used by SARS-CoV-2 to enter cells, thus providing an important new piece of information on SARS-CoV-2 biology [26]. Moreover, the reference Wuhan-Hu-1 strain motif 176LMDLE180, which is defined by the ELM resource as a clathrin box motif [19], is also located nearby. The clathrin box motif is found on cargo adapter proteins and interacts with the beta propeller structure located at the N-terminus of the clathrin heavy chain [27]. Perhaps since it is nearby, it also mimics some mammalian sequences or further enhances the connection with clathrin to improve penetration of the virus. Thus, the DPF motif probably plays a significant role in penetration of SARS-CoV-2 into the cell, and the absence of this sequence in the described variant may reduce its virulence.

Herein, we have described two SARS-CoV-2 sequences featuring unique mutations in the viral spike-protein gene. These mutations may change ACE2 receptor affinity, leading to changes in biological properties of the virus, such as pathogenicity or infectious activity.

## 5. Conclusions

As detailed above, we have described the identification of a new, previously-undescribed SARS-CoV-2 variant, which we have termed the *Northwest Variant* (NW variant). Taking into account significant features of the outer region of the S protein, it can be assumed that the biological properties of the NW variant may have significant differences from other variants. Therefore, the NW variant might potentially be a variant of concern (VOC). However, this assumption needs more rigorous study.

## Figures and Tables

**Table 1:** List of mutations observed in the NW variant of SARS-CoV-2.

Gene	NW Variant Strain of SARS-CoV-2					
	hCoV-19/Russia/SPb-117/2021(MW750605/EPI_ISL_1259282)			hCoV-19/Russia/Pskov-16/2021(MW750606/EPI_ISL_1259283)		
	Synonymous Substitution, nt	Nonsynonymous Substitution/Indel, nt	Substitution/Indel, aa	Synonymous Substitution/Indel, nt	Nonsynonymous Substitution/Indel, nt	Substitution/Indel, aa
5’ UTR				241C>T		
ORF 1a	**3037C>T** **5176A>G** **9070T>C** **9778C>T**	**1392C>T** **3281G>T** **3542A>G** **7005C>A** **10029C>T** **11451A>G** **12620T>A**	**S376L** **V1006F** **T1093A** **T2247N** **T3255I** **Q3729R** **S4119T**	**3037C>T** **5176A>G** **9070T>C** **9778C>T**	**1392C>T** **3281G>T** **3542A>G** **7005C>A** **10029C>T** **11451A>G** **12620T>A**	**S376L** **V1006F** **T1093A** **T2247N** **T3255I** **Q3729R** **S4119T**
ORF 1b	**17562G>T**	**14408C>T** 16934T>C **16985C>A** 17470C>A **19180G>T** **20759C>T**	**P314L** M1156T **T1173N** L1335I **V1905L** **A2431V**	**17562G>T**	**14408C>T** **16985C>A** **19180G>T** **20759C>T**	**P314L** **T1173N** **V1905L** **A2431V**
S gene	22882T>C	**21588C>T**	**P9L**	23449T>G	**21588C>T**	**P9L**
S1 domain		**Deletion** **21967_21993del** **22206A>G** **22296A>C** **23012G>A** **23403A>G**	**C136_Y144del** **D215G** **H245P** **E484K** **D614G**		**Deletion** **21967_21993del** **22206A>G** **22296A>C** **23012G>A** **23403A>G**	**C136_Y144del** **D215G** **H245P** **E484K** **D614G**
		**insertion** **23598_23599ins**	**N679delins** **KGIAL**		**insertion** **23598_23599ins**	**N679delins** **KGIAL**
S2 domain	**25000C>T**	**23900G>A** 24697G>T	**780E>K** 1045K>N	24370C>T 24721T>G **25000C>T**	**23900G>A**	**780E>K**
ORF 3a	**25603C>T** **26211G>T**	**25675T>A**	**L95M**	**25603C>T** **26211G>T**	**25675T>A**	**L95M**
M gene		**26568C>A**	**L16I**		**26568C>A** 27102G>A	**L16I** A194T
ORF 7a					27674A>G	Q94R
ORF 8	**28079G>T** **28271A>G**			**28079G>T** **28271A>G**		
N gene		**28881G>A** **28882G>A** **28883G>C**	**R203K** **G204R**		**28881G>A** **28882G>A** **28883G>C**	**R203K** **G204R**

Common mutations for NW strains hCoV-19/Russia/SPb-117/2021 and hCoV-19/Russia/Pskov-16/2021 are marked in bold.

## Data Availability

The authors confirm that the data supporting the findings of this study are available within the article [and/or] its supplementary materials.

## Supplementary Materials

The following are available online at https://www.mdpi.com/article/10.3390/v13061029/s1, Table S1: Primers used for near-complete genomic sequencing of SARS-CoV-2.
