# Supplementary material for: Characterization of a Novel SARS-CoV-2 Genetic Variant with Distinct Spike Protein Mutations
Source: Viruses. 2021 May 29;13(6):1029. doi: 10.3390/v13061029 (PMC8230012; doi:10.3390/v13061029)
Supplement: Supplementary file 1 [file viruses-13-01029-s001.zip › viruses-1236922-supplementary.pdf]

Table S1. Primers used for near-complete genomic sequencing of SARS-CoV-2.

Reg. No.	Pool	Left Primer Name	Left Primer Sequence	Right Primer Name	Right Primer Sequence	Product Length	Trimmed Overlap
1	1	CoV-2_1_L	ACCAACCAACTTTCGATCTCTTGT	CoV-2_1_R	TGTCTCACCACTACGACCGTAC	545	0
2	2	CoV-2_2_L	CATCAAACGTTCGGATGCTCGA	CoV-2_2_R	GGACATTCCCCATTGAAGGTGT	604	55
3	3	CoV-2_3_L	GCATGAAATTGCTTGGTACACGG	CoV-2_3_R	ACCTTCGGAACCTTCTCCAACA	632	62
4	4	CoV-2_4_L	GTTGGTTGCCATAACAAGTGTGC	CoV-2_4_R	ACACCACCTGTAATGTAGGCCA	576	50
5	1	CoV-2_5_L	AGGCCGCTATAACAATACTAGATGGA	CoV-2_5_R	TGTGGGAAGTGTTTCTCCCTCT	558	75
6	2	CoV-2_6_L	TCAAAGGGATTGTACAGAAAGTGTGT	CoV-2_6_R	AATCAATGCCCAGTGGTGTAAGT	554	62
7	3	CoV-2_7_L	TCGGTACAGAAGTAAATGAGTTCGC	CoV-2_7_R	TGGCTGCATTAACAACCACTGT	595	50
8	4	CoV-2_8_L	CCAGTTGTTCAGACTATTGAAGTGAATAG	CoV-2_8_R	CAGCGATCTTTTGTTCAACTTGCT	563	81
9	1	CoV-2_9_L	TCGCACAAATGTCTACTTAGCTGT	CoV-2_9_R	CGCAAATTCCAAGAAACAGTTCCA	622	58
10	2	CoV-2_10_L	AGAGGAGGCAAAGACAGTGCTT	CoV-2_10_R	CCATCTAGGTGGAATGTGGTAGGA	628	64
11	3	CoV-2_11_L	ACCATCTCACTTGCTGGTTCCT	CoV-2_11_R	AGCATCTTGTAGAGCAGGTGGA	586	87
12	4	CoV-2_12_L	ACTTCTATTAAATGGGCAGATAACAACTG	CoV-2_12_R	AGCACCGTCTATGCAATACAAAGT	562	50

13	1	CoV-2_13_L	CAGTATGAACTTAAGCATGGTACATTTACT	CoV-2_13_R	GGTTTTGTGCTCCAAAGACAACG	618	62
14	2	CoV-2_14_L	ACACACCCTCTTTTAAGAAAGGAGC	CoV-2_14_R	ACACCGTGTAACTATGTTAGTAGTTGT	558	81
15	3	CoV-2_15_L	GCTACTCATGGTTTAGCTGCTGT	CoV-2_15_R	TGCCAAAAACCACTCTGCAACT	625	63
16	4	CoV-2_16_L	GTGGTTTAGATTCTTTAGACACCTATCCT	CoV-2_16_R	ACTGTAGTGACAAGTCTCTCGCA	553	61
17	1	CoV-2_17_L	GCTTTTGCAAACTACACAATTGGAAT	CoV-2_17_R	CCTTGCCGAGCTGCTGAAATAA	593	52
18	2	CoV-2_18_L	ACGTACCAATGGAAAAACTCAAAACA	CoV-2_18_R	GCAGCAACAAAAAGGAACACAAGT	548	63
19	3	CoV-2_19_L	TGTTAATGTTGTAACAACAAAGATAGCACT	CoV-2_19_R	CGTGTGTCAGGGCGTAAACTTT	610	50
20	4	CoV-2_20_L	GCTGCTGAATGTACAATTTTTAAAGATGC	CoV-2_20_R	CCAGGTAAGAATGAGTAAACTGGTGT	564	61
21	1	CoV-2_21_L	TGAGGTTTAGAAGAGCTTTTGGTGA	CoV-2_21_R	AACCACTCTGCAAAACAGCTGA	604	63
22	2	CoV-2_22_L	TGTTGTCATCTCGCAAAGGCTC	CoV-2_22_R	AAGTCTGTGCCAGCATGAACTC	630	61
23	3	CoV-2_23_L	ATGGTTCATGTGGTAGTGTTGGT	CoV-2_23_R	GTAACAACCAGTGGTGTGTACCC	541	60
24	4	CoV-2_24_L	AGAAGATGAATTTACACCTTTTGATGTTGT	CoV-2_24_R	GCCCACATGGAAATGGCTTGAT	556	50
25	1	CoV-2_25_L	ATGGTGCTAGGAGAGTGTGGAC	CoV-2_25_R	CTGGACACATTGAGCCCACAAT	573	52
26	2	CoV-2_26_L	CCACTGTACAGTCTAAAATGTCAGATGT	CoV-2_26_R	GGGAACACAACCATCTCTTGCA	609	62

27	3	CoV-2_27_L	GGGCAAAAGTTACTAGTGCTATGCA	CoV-2_27_R	CCTACAAGGTGGTTCCAGTTCTG	579	62
28	4	CoV-2_28_L	GGAGGTAGGTTTGTACTTGCACTG	CoV-2_28_R	CCCACAGGGTCATTAGCACAAG	558	66
29	1	CoV-2_29_L	CTGTACTGCCGTTGCCACATAG	CoV-2_29_R	AGCATAGACGAGGTCTGCCATT	585	59
30	2	CoV-2_30_L	GCTGTTGCTAAACATGACTTCTTTAAGT	CoV-2_30_R	TGGGTGGTATGTCTGATCCCAA	607	57
31	3	CoV-2_31_L	CAAAGCCTTACATTAAGTGGGATTTGT	CoV-2_31_R	GCAGCATTACCATCCTGAGCAA	563	54
32	4	CoV-2_32_L	ACAAAGACTTCTATGACTTTGCTGTGT	CoV-2_32_R	AACATGTTGTGCCAACCACCAT	560	50
33	1	CoV-2_33_L	ACTATGACCAATAGACAGTTTCATCAAAAA	CoV-2_33_R	AGCCACTAGACCTTGAGATGCA	640	60
34	2	CoV-2_34_L	TTTGTGAATGAGTTTTACGCATATTTGC	CoV-2_34_R	AGCCTGTAAGACTGTATGCGGT	580	60
35	3	CoV-2_35_L	GCTACATGATGAGTTAACAGGACACA	CoV-2_35_R	CTCTGTCAGACAGCACTTCACG	612	81
36	4	CoV-2_36_L	TCAAGCTTTTTGCAGCAGAAACG	CoV-2_36_R	TCACATAGTGCATCAACAGCGG	569	50
37	1	CoV-2_37_L	GGTAAGAGTCATTTTGCTATTGGCC	CoV-2_37_R	TGCAGATGAAACATCATGCGTGA	604	50
38	2	CoV-2_38_L	CACTGTGAGTGCTTTGGTTTATGAT	CoV-2_38_R	TCATGTCCTTAGGTATGCCAGGT	603	63
39	3	CoV-2_39_L	TGGGTTACATCCTACACAGGCA	CoV-2_39_R	CATAGACAACAGGTGCGCTCAG	583	55
40	4	CoV-2_40_L	CTCTCTGACAGAGTCGTATTTGTCTT	CoV-2_40_R	GTCACTACAAGGCTGTGCATCA	547	52

41	1	CoV-2_41_L	CCCAGTTCTTCACGACATTGGT	CoV-2_41_R	AAACCCACAAGCTAAAGCCAGC	555	52
42	2	CoV-2_42_L	ACGTTGCAATTTAGGTGGTGCT	CoV-2_42_R	AGAACACCATTACGGGCATTTCT	583	62
43	3	CoV-2_43_L	AGCCAAGAAACCAACTGAAACGA	CoV-2_43_R	TGAACCTGTTTGCGCATCTGTT	542	58
44	4	CoV-2_44_L	TGATTGGACTAGCTAAACGTTTTAAGGA	CoV-2_44_R	AAACAGCTGTACCTGGTGCAAC	541	59
45	1	CoV-2_45_L	TGAATGTCGCAAAATATACTCAACTGTG	CoV-2_45_R	AACCATCTATTTGTTCGCGTGGT	541	78
46	2	CoV-2_46_L	GCATGGTGGACAGCCTTTGTTA	CoV-2_46_R	ACCTCTTAGTACCATTGGTCCCA	579	57
47	3	CoV-2_47_L	TCCTCAGTTTTACATTCAACTCAGGA	CoV-2_47_R	TGGTTCTAAAGCCGAAAAACCCT	544	53
48	4	CoV-2_48_L	AGGGAATTTGTGTTTAAGAATATTGATGGT	CoV-2_48_R	ACTTAAAAGTGGAAAATGATGCGGAA	569	54
49	1	CoV-2_49_L	TAACGCCACCAGATTTGCATCT	CoV-2_49_R	TAGGTCCACAAACAGTTGCTGG	558	61
50	2	CoV-2_50_L	GGTTTCCAACCCACTAATGGTGT	CoV-2_50_R	TGACTAGCTACACTACGTGCCC	585	51
51	3	CoV-2_51_L	GCTGAACATGTCAACAACTCATATGA	CoV-2_51_R	TGATGAAGCCAGCATCTGCAAG	548	64
52	4	CoV-2_52_L	AGTCAAACAAATTTACAAAACACCACCA	CoV-2_52_R	TGCACTTCAGCCTCAACTTTGT	625	101
53	1	CoV-2_53_L	TGCACAAGCTTTAAACACGCTT	CoV-2_53_R	TGAGTCTAATTCAGGTTGCAAAGGA	578	66
54	2	CoV-2_54_L	ACCACAAATCATTACTACAGACAACACA	CoV-2_54_R	TGCAGTAGCGCGAACAAAATCT	598	55

55	3	CoV-2_55_L	AGGAGTCAAATTACATTACACATAAACGAA	CoV-2_55_R	GACTTGTTGTGCCATCACCTGA	566	79
56	4	CoV-2_56_L	GCCAACTATTTTCTTTGCTGGCA	CoV-2_56_R	ACACAATCGAAGCGCAGTAAGG	547	60
57	1	CoV-2_57_L	CGTTTCGGAAGAGACAGGTACG	CoV-2_57_R	AATGACCACATGGAACGCGTAC	603	64
58	2	CoV-2_58_L	TGGATCACCGGTGGAATTGCTA	CoV-2_58_R	TCAATCTCCATTGGTTGCTCTTCA	640	68
59	3	CoV-2_59_L	TTATGAGGACTTTTAAAGTTTCCATTTGGA	CoV-2_59_R	TGTTCGTTTAGGCGTGACAAGT	642	75
60	4	CoV-2_60_L	TTTGTGCTTTTTAGCCTTTCTGCT	CoV-2_60_R	ACTGCCAGTTGAATCTGAGGGT	568	63
61	1	CoV-2_61_L	AGAGTATCATGACGTTCGTGTTGT	CoV-2_61_R	GAACGAGAAGAGGCTTGACTGC	612	85
62	2	CoV-2_62_L	GCAATCCTGCTAACAATGCTGC	CoV-2_62_R	CTGTGTAGGTCAACCACGTTCC	559	67
63	3	CoV-2_63_L	CATTGGCCGCAAATTGCACAAT	CoV-2_63_R	TAGGCAGCTCTCCCTAGCATTG	621	61
64	4	CoV-2_64_L	TGGATGACAAAGATCCAAATTTCAAAGA	CoV-2_64_R	AAAATCACATGGGGATAGCACTACT	561	451
